# Massive Gastrointestinal Hemorrhage Secondary to Typhoid Fever

**DOI:** 10.7759/cureus.17552

**Published:** 2021-08-30

**Authors:** Sameen Aamer, Sara Ahmed, Khakan Ahmed, Nadeem Iqbal

**Affiliations:** 1 Internal Medicine, Shifa International Hospital Islamabad, Islamabad, PAK; 2 Medicine, Shifa International Hospital Islamabad, Islamabad, PAK; 3 Gastroenterology, Shifa International Hospital Islamabad, Islamabad, PAK

**Keywords:** typhoid fever, massive gastrointestinal hemorrhage, endoscopic clipping, salmonella typhi, colonoscopy, gastrointestinal complications

## Abstract

Typhoid fever is caused by *Salmonella typhi*, a gram-negative organism. The disease usually presents with high-grade fever, abdominal pain, and diarrhea. Gastrointestinal hemorrhage is a frequent complication of the disease. However, adequate treatment with antibiotics has lowered the rate of complications. We present the case of a 21-year-old male who was admitted to the hospital with high-grade fever and per rectal bleeding. A few hours after admission, the patient had episodes of massive per rectal bleeding which resulted in hemodynamic instability. The bleeding was then successfully controlled with endoscopic hemoclipping. Concurrently, his blood culture results showed growth of *Salmonella typhi* for which antibiotic therapy was initiated, and the patient's condition improved thereafter. This report highlights the rare occurrence of massive lower gastrointestinal bleeding in patients with typhoid fever. It also signifies the use of endoscopic therapy with endoclips for the management of massive lower gastrointestinal bleeding.

## Introduction

Typhoid fever is caused by *Salmonella typhi*, an enteroinvasive gram-negative bacteria [1]. The most common presenting symptoms of the disease include fever, generalized abdominal pain, diarrhea, anorexia, and weight loss. Other rare complications of typhoid fever are gastrointestinal hemorrhage, bowel perforation, peritonitis, myocarditis, and endocarditis. However, timely and effective treatment with antibiotics has reduced the incidence of complications of typhoid fever [1].

Gastrointestinal (GI) bleeding occurs in 10% of affected people [2]. The bleeding is usually mild, but massive GI bleeding occurs in 2% of patients [3]. GI bleeding can be managed either conservatively, surgically, or endoscopically [4,5]. Herein, we report a case of a 21-year-old male who initially presented with massive gastrointestinal bleeding, and was later diagnosed with typhoid fever, to highlight this rare complication of the disease. This case also highlights that in prevalent areas enteric fever should be considered in the differential diagnosis in patients, especially those presenting with fever, abdominal pain, and diarrhea, and early treatment should be initiated to prevent severe complications like GI bleed. 

## Case presentation

A 21-year-old male from Peshawar, Pakistan, presented to the emergency department with a high-grade fever associated with rigors and chills for 10 days. He also had nausea, vomiting, and constipation followed by diarrhea for seven days, along with per rectal bleeding for two days.

Initially, he had foul-smelling diarrhea only, but later, he developed episodes of rectal bleeding consisting of fresh blood mixed with stool. He had not had any similar symptoms previously. He had no history of sick contacts and none of the family members had similar complaints. The patient had no history of weight loss, recent travel, contact with poultry, drug abuse, or any recent antibiotic use. He had no past medical history. He had no family history of tuberculosis, gastrointestinal illnesses, or malignancies. He consulted a local doctor when the symptoms initially appeared, and he was treated with an antipyretic, antiemetic, and oral tablet metronidazole 400 mg thrice a day.

On examination, his vitals showed: blood pressure 100/70 mmHg, pulse 110/min, temperature 104 ℉, respiratory rate 20/min, and oxygen saturation 98%. He had pallor but no jaundice. His abdomen was soft and tender in the periumbilical region. There was no rebound tenderness and the rest of the systemic examination was unremarkable. His outside workup is shown in Table 1.

**Table 1:** Patient's laboratory tests done at the local hospital WBC, white blood cell; ALT, alanine transaminase; ALP, alkaline phosphatase; INR, international normalized ratio; ESR, erythrocyte sedimentation rate; Urine R/E, urine routine examination; HIV, human immunodeficiency virus; CT, computed tomography.

Test	Results
Hemoglobin (g/dL)	14.97
WBC (x 10^3^)	7.41
Platelet count (x 10^3^)	195
ALT (U/L)	159
ALP (U/L)	144
Total Bilirubin (mg/dL)	0.46
Serum Sodium (mmol/L)	131
Serum Potassium (mmol/L)	4.01
INR	1.1
Amylase (U/L)	184
Albumin (g/dL)	2.88
ESR (mm/hour)	23
Urine R/E	Albumin 1+ , Leukocyte 1+
Malarial Parasite Smear	Not Seen
Hepatitis A Virus	Negative
Hepatitis C Virus	Negative
Hepatitis B Surface Antigen	Negative
HIV	Negative
CT scan abdomen & pelvis	Significantly thickened terminal ileum, involving the ileocecal junction, and multiple large nodes

A few laboratory results were repeated in the emergency department and the results are displayed in Table 2.

**Table 2:** Investigations performed in the emergency department WBC, white blood cell; CRP, c-reactive protein; INR, international normalized ratio.

Test	Result
Hemoglobin (g/dL)	10.9
WBC (x 10^3^)	9.51
Platelet count (x 10^3^)	524
CRP (mg/L)	55
Ionized calcium (mmol/L)	4.0
Creatinine (mg/dL)	0.6
Urea (mmol/L)	6.42
INR	1.06
Random blood sugar (mg/dL)	107

In the emergency department, his blood sample was taken for culture and sensitivity, and then he was given an intravenous antibiotic (ceftriaxone), a proton pump inhibitor, and tranexamic acid.

The patient was then admitted by the gastroenterology team with a provisional diagnosis of enteric fever complicated with lower gastrointestinal bleed. He was admitted to the floor in a vitally stable condition, without any active bleeding. He had one episode of per rectal bleed three hours later but remained vitally stable. Then after another hour, he started having massive bleed. On examination, he looked pale and toxic. Vitals recorded at that time showed: blood pressure 90/60 mmHg, pulse 125/min, temperature 98℉, and oxygen saturation 97% at room air. The chest was bilaterally clear on auscultation. He was reassessed by the gastroenterology team and considering the patient's condition the rapid response team was called. The patient was given an intravenous bolus of normal saline and 1g of injection tranexamic acid.

The patient was then shifted to the medical step-down (MSD) unit, where he was started on thrice daily intravenous injections of meropenem 1 g, and tranexamic acid 1 g. His repeat hemoglobin level was 8.4 g/dL, therefore three units of packed red blood cells were transfused. The surgery team was consulted and the patient was prepped with Kleen enema, for flexible sigmoidoscopy. Eventually, a colonoscopy was performed.

Colonoscopy findings showed scattered fresh and clotted blood throughout the colon. However, flushing revealed normal colonic mucosa till the ascending colon. In the caecum, there were multiple, variable-sized, deep, and superficial ulcers with surrounding erythema and edema, but without any active bleeding. At the terminal ileum, there was one 3 cm large, deep ulcer with a large visible vessel (Forrest 2-A). This was identified as the likely source of bleeding. There were two to three more ulcers at the terminal ileum, but they were comparatively small.

During the procedure, two endoscopic clips were applied to visible blood vessels in the ulcers, and hemostasis was achieved. Biopsies were also taken from the ulcers present in the caecum and other parts of the colon. After colonoscopy results, the patient was suspected to have typhoid, tuberculosis, or cytomegalovirus (CMV) infection.

The patient remained stable post-colonoscopy. His hemoglobin remained stable at 10.5 g/dL and he had no further episodes of per rectal bleed. It was planned that in case of any further episodes of per rectal bleed, interventional radiology-guided embolization would be performed.

Soon, his culture results were available. Stool culture showed no growth. Results of his blood culture are shown in Table 3, which showed growth of *Salmonella typhi* sensitive to ceftriaxone and cefixime only. Therefore, injection meropenem was stopped, and injection ceftriaxone 2 g, twice a day, intravenously, was initiated.

**Table 3:** Patient's blood culture and sensitivity report C/S, culture and sensitivity; MDR, multiple drug resistance; R, resistance; S, susceptibility; I, intermediate resistance.

BLOOD C/S (ADULT): SALMONELLA TYPHI (MDR) Isolated	
Antibiotics	SALMONELLA TYPHI (MDR)
Ampicillin	R
Cefixime	S
Ceftriaxone	S
Chloramphenicol	R
Ciprofloxacin	I
Co-Trimoxazole	R

The patient was then shifted back to the floor after two days. He remained hemodynamically stable. He was monitored for another four days and was discharged then. His hemoglobin at discharge was 11.8 g/dL. The patient was advised to continue injection ceftriaxone 2 g, twice daily, intravenously, for a total of 14 days. A follow-up after two weeks was scheduled, when biopsy results would be reviewed.

The patient visited the gastroenterology clinic, after two weeks. His blood test showed a hemoglobin of 13.1 g/dL. The colon biopsy showed mild active colitis only and was negative for inflammatory bowel disease, granulomatous inflammation, or malignancy. The patient had completed his 14-day course of injection ceftriaxone, was afebrile, and had no complaint of per rectal bleed.

## Discussion

Typhoid fever is caused by a gram-negative bacteria, *Salmonella typhi*, and is mainly a disease of the developing world [1,6]. As per global estimates, approximately 600,000 deaths occur annually due to enteric fever, and most of them occur in developing countries, including Pakistan [7]. The disease is transmitted via the fecal-oral route, and ingestion of water contaminated with the organism is the most common source of infection [4,8]. After ingestion, the organism enters the small bowel mucosa via the regional lymphatic and hematogenous system and replicates in the reticuloendothelial system. This most commonly occurs at the terminal ileum due to a large number of Peyer's patches at that site [8]. The disease then manifests with high-grade fever, generalized abdominal pain, diarrhea, weight loss, and decreased appetite. Other uncommon presentations of typhoid fever include pneumonia, orchitis, osteomyelitis, Guillain-Barre syndrome, endocarditis, and myocarditis. Gastrointestinal hemorrhage, bowel perforation, pancreatitis, and cholecystitis are rare GI complications of the disease [2]. Complications are highly associated with multiple drug-resistant (MDR) enteric fever than with drug-sensitive enteric fever. However, the mortality rates are almost equal for both [9]. 

Blood culture is the gold standard test for the diagnosis of typhoid fever [10]. A positive blood culture confirms the disease. After diagnosis, the appropriate treatment is initiated. Supportive treatment including hydration, antipyretics, and adequate nutrition is started. First-line drugs for the treatment of typhoid fever are chloramphenicol, ampicillin, and trimethoprim-sulfamethoxazole [11]. For MDR typhoid fever (typhoid not susceptible to the first-line drugs), third-generation cephalosporins are the drugs of choice. Other alternatives include ciprofloxacin [11,12]. The excessive use of antibiotics has now led to the evolution of extensively drug-resistant (XDR) typhoid fever, which is only susceptible to azithromycin [13]. 

Timely diagnosis and treatment with antibiotics have reduced the complications of typhoid fever [1]. However, in patients with a prolonged disease course, or those not diagnosed and treated timely, the bacteria multiplies and invades the submucosa, causing ulcers that can perforate or erode a vessel leading to hemorrhage. Gastrointestinal bleeding occurs in the second and third weeks of the disease [1]. It occurs in 10% of the cases [2]. The incidence rate of bleeding is 12.5% [1]. The most commonly involved sites are the terminal ileum (100%), followed by the ileocecal valve (57%), the ascending colon (43%), and the transverse colon (29%). Thus, the most common site of intestinal bleeding in typhoid fever is the ileum. [14]

Colonoscopy is the gold standard test for diagnosing lower GI bleeding [15]. The most common colonoscopic finding in complicated typhoid fever is multiple variable-sized, punched-out ulcers with raised margins [14].

Approximately 90% of cases of mild gastrointestinal bleeding are managed conservatively [1]. Severe lower GI bleeding occurs in 2% of cases which can cause morbidity and mortality [3]. Massive bleeding requires surgical intervention, exploratory laparotomy, right hemicolectomy, or segmental resection [4,14]. However, due to recent advances like endotherapy and angioembolization, the need for surgery has reduced significantly [5].

Endoscopic techniques are now considered the standard in managing GI bleeding; this includes adrenaline injection, thermal coagulation, and hemoclipping. These options can be used either alone or as a combination, to obtain an effective response [15,16]. Endoclips are efficacious if there is active vascular bleeding, or non-bleeding visible vessels and clots [17]. The effectiveness of hemoclipping in controlling massive colon bleeding in patients with ulcerative colitis has also been reported by Yoshida et al [18]. Moreover, endoclipping is also superior to adrenaline injection and electrocoagulation because it reduces the risk of recurrent bleeding and thermal injury [19]. Therefore, due to advancements in endoscopic treatment, patients can recover successfully [3].

This case brings to light the rare occurrence of massive gastrointestinal bleeding in patients affected with *Salmonella typhi.* Moreover, it also illustrates that typhoid fever should be considered in the provisional diagnosis of massive lower gastrointestinal bleeding, especially in patients with a history of recent travel to or those who reside in areas of greater prevalence-mainly South or South East Asia. Typhoid fever is a common cause of morbidity in the developing world [20]. Therefore, early suspicion and initiation of treatment can be pivotal in the reduction of mortality and morbidity.

This study also reports the favorable outcomes of endoscopic therapy in typhoid-ulcer-related gastrointestinal bleeding. The literature available regarding the effectiveness of endoscopic therapy for massive gastrointestinal hemorrhage secondary to typhoid fever is not sufficient and very few cases are reported worldwide [2].

## Conclusions

Considering the increased prevalence of typhoid fever, especially in developing countries, the disease burden should be decreased by creating awareness about hygiene and by making clean drinking water accessible to all. Furthermore, the disease should not be overlooked; timely and appropriate treatment should be provided to prevent complications. Even though antibiotic use has significantly reduced the risk of complications, this rare cause of gastrointestinal bleeding should be considered in the differential diagnosis. Moreover, prompt risk stratification, such as by using the Oakland score, can be considered by the physicians to prevent the occurrence of massive lower GI bleed during hospital admission. 
